# Home-related and work-related injuries in Makwanpur district, Nepal: a household survey

**DOI:** 10.1136/injuryprev-2020-043986

**Published:** 2020-11-04

**Authors:** Puspa Raj Pant, Toity Deave, Amrit Banstola, Sumiksha Bhatta, Elisha Joshi, Dhruba Adhikari, Sunil Raja Manandhar, Sunil Kumar Joshi, Julie A Mytton

**Affiliations:** 1 Faculty of Health and Applied Sciences, University of the West of England, Bristol, UK; 2 Kathmandu Medical College Public Limited, Nepal Injury Research Centre, Kathmandu, Nepal; 3 Mother and Infant Research Activities (MIRA), Kathmandu, Nepal; 4 Department of Paediatrics, Kathmandu Medical College Public Limited, Kathmandu, Nepal; 5 Department of Community Medicine, Kathmandu Medical College Public Limited, Kathmandu, Nepal

**Keywords:** public health, cross sectional study, outcome of injury, community, costs, surveys

## Abstract

**Objective:**

To describe the epidemiology of home-related and work-related injuries, their mechanisms, inequalities and costs associated with these injuries.

**Methods:**

A household survey was undertaken in three palikas of Makwanpur district between April and June 2019. Data were collected electronically on non-fatal injuries that occurred in the previous 3 months and fatal injuries that occurred in the previous 5 years.

**Findings:**

17 593 individuals were surveyed from 3327 households. Injury rates were 8.0 per 1000 population for home injuries and 6.4 per 1000 for work-related injuries; 61.0% of home injuries were among women and 69.9% of work-related injuries among men. Falls were the cause of 48% home injuries, affecting 50.9% of men and 46.5% of women. Burns/scalds were higher in women than men, affecting 17.4% of women reporting home injuries. Cuts and piercings accounted for 39.8% of all work-related injuries and 36.3% were falls. Injury incidence varied by ethnic group: home injuries were highest in Brahmin (12.0 per 1000) and work-related injuries highest in Rai groups (21.0 per 1000). The total mean costs (transport and treatment) of work-related injury was US$143.3 (SD 276.7), higher than for home injuries (US$130.4, SD 347.6). The number of home (n=74, 64.9%) and work-related (n=67, 77.9%) injuries were higher in families below the poverty line than families in the next income bracket (home: n=22, 19.3%; work: n=11, 12.8%).

**Conclusions:**

Home-related and work-related fall injuries are common. The inequalities in injury identified in our study by rurality, age, sex, income level and ethnic group can help target injury prevention interventions for vulnerable groups.

## Introduction

Injury is relatively new on the public health agenda for Nepal. Hospital-based reports provide some information on injury-related mortality and morbidity, but community-based information derived from nationally representative samples is limited. Analysis of nationally representative census (2001) data reported an injury mortality rate of 30 per 100 000 population^1^ whereas the corresponding estimate for Nepal, produced by the Global Burden of Disease (GBD) study in 2017 was 56.3 per 100 000 population.^2^ In the absence of a robust death registration system in Nepal to record underlying causes of death, modelled data (such as those from the GBD study) are likely to provide the most robust estimates of injury deaths. Nepal has the second highest per 100 000 injury death rate among those estimated for five nearby South Asian countries: Bangladesh (36.8), Bhutan (38.3), India (70.8), Nepal (56.3) and Pakistan (53.9).^2^ Nepal’s Health Management Information System is the only routinely collected source of information on non-fatal injuries; this recorded 1.1 million outpatient department visits for injuries in the year 2017–2018.^3^ The GBD study estimated 1.5 million injury outpatient visits in 2017, an annual rate of 50 per 1000 population.^2^ Household (HH) surveys conducted in different parts of Nepal at different times have found different estimates for non-fatal injuries; for example, in urban Dharan, minor injuries occurred in 35 per 1000 population (1 month recall) and major injuries in 7 per 1000 population (1 year recall).^4^ In rural Bhaktapur, the general injury rate was 29 per 1000.^5^ Minor injuries were defined as those resulting in a loss of less than 30 days of usual activity and major injuries as those resulting in a loss of 30 or more days. One study, conducted in rural eastern Nepal, reported an incidence of minor non-fatal injuries of 31 per 1000 population^6^; a nationwide survey, which used a lifetime experience of an injury, reported a high incidence of 131 per 1000 population non-fatal injuries.^7^ In Makwanpur, the incidence rate for injuries among children aged 0–17 years was 25 per 1000.^8^ The incidence of non-fatal home injuries among children aged less than 5 years living in rural areas of Makwanpur district was found to be as high as 232 per 1000.^9^ The different estimates of injury incidence use different types of data and varied definitions of injury in diverse population groups and settings. These sources identify the place of injury but not whether they were home or work related, nor have they reported on costs associated with injuries. A recent systematic review of injury research in Nepal identified an absence of evidence about inequalities in injury occurrence.^10^

This paper describes a study to explore the epidemiology of injuries related to home and work activities, inequalities in injury incidence and costs associated with injuries.

## Methods

This study used a cross-sectional, community-based survey where data on injuries and the reported impact of these injuries on participants were collected face-to-face by trained data collectors between April and June 2019. The setting was the Makwanpur district of Nepal, which includes three distinct administrative areas (known as 'palika') that has a topography representative of the country; research conducted there has the potential to be applicable to other districts.

A sample size of 3325 was calculated using the standard formula suggested in guidelines provided by the United Nations for conducting community-based HH surveys in low-income countries: a multistage, cluster sampling methodology was applied to selected HHs as the survey unit.^11^ The three palikas were selected purposively: one rural municipality, one municipality and one sub-metropolitan city. In each palika, four non-adjacent wards were selected purposively. The selection of HHs in the wards was proportional to the total number of HHs in each palika, systematic random sampling was used. All identified, eligible HHs were invited to take part. If there was no reply from a HH at initial contact, the HH was visited again at a different time of day. If unsuccessful at the second attempt, the next HH on the list was selected.

For each HH there was one respondent; the inclusion criteria for this person was that they were 18 years or over and, if a child was reported to have been injured, they were the main caregiver. Inclusion criteria for those reported to have been injured was that they could be any age but were, ordinarily, resident in one of the three palikas. Data were collected electronically on handheld tablet computers using REDCap data capture software by trained data collectors. The data collected included information about the HH, about non-fatal injuries that had occurred in the previous 3 months and fatal injuries that had occurred in the previous 5 years, including type, location, circumstances and consequences of the injury sustained. Data collection tools were adapted from the WHO guidelines on conducting community surveys on injuries and violence.^12^

An ‘injury’ was defined as an incident that resulted in a loss of at least 1 day of usual activity (eg, absence from school) or one that required medical attention. A ‘home injury’ was defined as any injury that occurred within and around the home, not related to paid work or trade. ‘Work-related injury’ was defined as any injury that occurred while working in a paid job or for family subsistence, whether that work occurred at home, during a journey related to work or at a workplace, such as an office or factory. To collect data about ethnic groups, HHs were offered over 100 different ethnic groups to choose from. When describing ethnicity, we report the risks for the six most commonly reported ethnic groups and ‘other’.

The data were cleaned, coded and analysed using IBM SPSS Statistics V.26. Descriptive statistics, percentages, rates and costs associated with injuries were calculated. For normally distributed data, the mean and SD are presented, the median and the IQR for skewed data. Differences between groups were investigated using non-parametric tests (Χ^2^). To ensure statistical disclosure control, cell numbers of less than five have been removed and, where necessary, for example, table 2, they are described in percentages. We used Nepal Census population size for ethnicities in the Makwanpur district to obtain denominator population for calculating injury incidence rates. Out-of-pocket (OOP) expenditure was the payment made by the HH from its primary income or savings to cover the costs of injury (ie, treatment costs and transportation costs). If payments were made by other methods (eg, borrowed money), they were deducted from the total costs of injury in order to calculate OOP expenditure.

**Table 2:** Sociodemographic characteristics of home and work-related injuries by gender

	Home injuries			Work-related injuries		
	Female row (%)	Male row (%)	Total col (%)	Female row (%)	Male row (%)	Total col (%)
**Palikas**						
Bakaiya	70.0	30.0	21.3	39.6	60.4	42.5
Hetauda	60.0	40.0	63.8	21.6	78.4	32.7
Thaha	52.4	47.6	14.9	25.0	75.0	24.8
**Total**	**61.0**	**39.0**	**100**	**30.1**	**69.9**	**100**
**Age groups**						
Infants	*	*	1.4	*	*	0.0
1–14 years	40.4	59.6	33.3	*	71.4	6.2
15–24 years	80.0	*	7.1	42.9	57.1	18.6
25–44 years	80.6	19.4	25.5	17.1	82.9	36.3
45–64 years	67.7	32.3	22.0	36.1	63.9	31.9
65 years or over	53.3	46.7	10.6	*	62.5	7.1
**Total**	**61.0**	**39.0**	**100**	**30.1**	**69.9**	**100**
Economic activity*						
Mainly unemployed	54.5	45.5	8.7	*	66.7	8.0
Employed salaried	*	*	4.7	*	87.5	14.3
Home-maker	92.1	*	29.9	60.0	40.0	22.3
Agriculture	42.9	57.1	11.0	35.0	65.0	17.9
Student	55.0	45.0	31.5	33.3	66.7	13.4
Wage earners	*	*	0.0	*	100.0	14.3
All other occupation	66.7	33.3	14.2	*	81.8	9.8
**Total**	**66.1**	**33.9**	**100**	**30.4**	**69.6**	**100**
**Ethnicity**						
Tamang	60.0	40.0	24.8	27.3	72.7	19.5
Brahmin (Hill)	66.7	*	8.5	31.3	68.8	14.2
Newar	*	*	4.3	*	*	0.0
Chhetri	72.7	*	7.8	*	*	2.7
Kami	*	*	2.8	*	*	6.2
Rai	60.0	40.0	39.0	32.7	67.3	43.4
All other ethnicities	55.6	44.4	12.8	*	87.5	14.2
**Total**	**61.0**	**39.0**	**100**	**30.1**	**69.9**	**100**
**Mechanism of injury**						
Road traffic injury	*	*	0.0	*	100.0	7.1
Fall (including push or jump)	58.8	41.2	48.2	31.7	68.3	36.3
Poisoning	*	*	2.1	*	*	0.0
Animal/insect or reptiles’ bite/sting	75.0	*	8.5	*	*	2.7
Fire, burn or scald	75.0	25.0	14.2	*	*	1.8
Electrocution	*	*	0.7	*	*	1.8
Cut, pierce or impale	47.8	52.2	16.3	42.2	57.8	39.8
Injured by a blunt object	72.7	*	7.8	*	91.7	10.6
Other	*	*	0.0	*	100.0	7.1
**Total**	**58.8**	**41.2**	**48.2**	**31.7**	**68.3**	**36.3**
**Activity during injury**†						
Leisure or playing	53.2	46.8	55.0	*	*	0.0
Work	*	*	0.0	31.3	68.8	85.0
Other activities	72.1	27.9	43.6	*	*	0.9
Education	*	*	0.7	*	*	0.0
Travelling to and from work or school	*	*	0.0	*	80.0	8.8
Travelling for other purpose	*	*	0.7	*	*	5.3
**Total**	**61.4**	**38.6**	**100**	**30.1**	**69.9**	**100**

Total usable sample size=254 individuals. Cells with less than five observations removed.

*Information for 15 people is not available.

†One person declined to answer.

## Results

A total of 3327 HHs were surveyed within the three palikas: Bakaiya (499 HHs; 15.0%), Hetauda (2,256 HHs; 67.8%) and Thaha (572 HHs; 17.2%), including 17 593 individuals (49.5% women) (table 1). Seven HHs declined to participate in the survey. Within the previous 3 months, 358 (10.8%) HHs reported that someone had been injured. Bakaiya reported the highest proportion (n=107, 21.4%) of survey HHs with an injured person. In contrast, the percentage of injured householders in Hetauda and Thaha was 8.3% (n=187) and 11.2% (n=64), respectively.

**Table 1:** Rates of home and work-related injuries in the sample population

Category (whole sample—injured and uninjured)	Home injuries n (rate per 1000)	Work injuries n (rate per 1000)
**Gender**		
Female (n=8704)	86 (9.9)	34 (3.9)
Male (n=8889)	55 (6.2)	79 (8.9)
		Χ^2^=0.513, p>0.05†
**Palika**		
Bakaiya (n=3023)	30 (9.9)	48 (15.9)
Hetauda (n=11 485)	90 (7.8)	37 (3.2)
Thaha (n=3085)	21 (6.8)	28 (9.1)
		Χ^2^=37.112, p<0.001†
**Age groups**		
Infants (n=258)	*	*
1–14 years (n=3738)	47 (12.6)	7 (1.9)
15–24 years (n=3662)	10 (2.7)	21 (5.7)
25–44 years (n=5617)	36 (6.4)	41 (7.3)
45–64 years (n=3146)	31 (9.9)	36 (11.4)
65+ years (n=1172)	15 (12.8)	8 (6.8)
**Family size**		
Small family (up to 4 members) (n=7512)	42 (5.6)	35 (4.7)
Medium family (5–8 members) (n=8585)	82 (9.6)	65 (7.6)
Large family (9 or more members) (n=1496)	17 (11.4)	13 (8.7)
		Χ^2^=16.923, p<0.001†
Ethnicity*		
Rai (n=334)	*	7 (21.0)
Brahmin (Hill) (n=2481)	35 (14.1)	22 (8.9)
Kami (n=510)	*	*
Chhetri (n=1882)	12 (6.4)	16 (8.5)
Tamang (n=8409)	55 (6.5)	49 (5.8)
Newar (n=1091)	11 (10.1)	*
All other ethnicities (n=2885)	18 (6.2)	16 (5.5)
**Total (N=17 593**)	**141** (**8.0**)	**113** (**6.4**)
		Χ^2^=16.923, p<0.001†

Cells with less than five observations removed.

*Estimated number of people for ethnicity based on the proportion of census data 2011.

†Post hoc analyses to test statistical difference in rates for both injury types combined.

Of the 394 reported injuries, 254 (64.5%) met the criteria for home or work-related injuries. There were 141 home-related injuries (8.0 per 1000 population) and 113 work-related injuries (6.4 per 1000 population). Sixty-one fatal injuries, due to any cause, were reported to have occurred over the previous 5 years, two of which were during the 3-month data collection period. Due to the low incidence, these cases are not reported further in this paper.

The age range of persons sustaining non-fatal injuries was between <1 and 87 years with a median of 35 years (IQR 34) (table 1). The median age of injured men was 35 years (IQR 37) and 37.5 years (IQR 32) for women. Men had a slightly higher rate of injury (15.1 per 1000) compared with women (13.8 per 1000), however, home injuries were higher among women (9.9 per 1000) than men and work-related injuries were higher among men (8.9 per 1000) compared with women. Within age groups, children aged 1–14 years and adults aged 45–64 years had the highest home injury rates (12.6 and 12.8 per 1000, respectively) whereas people aged 45–64 years had the highest work-related injuries (11.4 per 1000). Tamang were the largest ethnic group in the areas surveyed. Two ethnic groups, Rai (21.0 per 1000) and hill-living Brahmin (12.0 per 1000) reported the highest rates of work-related and home injuries, respectively (table 1). Post hoc analyses found that there was a statistical difference in rates for both injury types between different ethnic groups (Χ^2^=16.923, p<0.001) and between smaller and larger families (Χ^2^=25.175, p<0.001) (table 1). Large families (9+ members) had higher rates of both home injuries (11.4 per 1000) and work-related injuries (8.7 per 1000 population) compared with small (1–4 members) and medium sized (5–8 members) families (table 1).

This study recorded 141 individuals (8.0 per 1000) with home-related injuries and 113 (6.4 per 1000) with work-related injuries. Injury incident rates for women were higher (9.9 per 1000) for home injuries compared with work-related injuries (3.9 per 1000); this was true in all three palikas. Work-related injury rates were higher among men (8.9 per 1000) compared with home injuries (6.2 per 1000). In rural Bakaiya, both the rate of home injuries (9.9 per 1000) and work-related injuries (15.9 per 1000) were higher than in Hetauda (urban) and Thaha (suburban) (table 1). Post hoc analyses found a statistical difference in rates for both injury types between different palikas (Χ^2^=37.112, p<0.001).

Among all home injuries, 45.4% occurred in children (<16 years) and the elderly (65+ years) whereas 86.7% of all work-related injuries were among people aged 15–64 years. Women reported more injuries at home than men at all ages except for children (<16 years) where boys had proportionally higher numbers of injuries than girls (n=19, 40.4% and n=28, 59.6%, respectively). For work-related injuries, men had, proportionally, a much higher number of injuries than women in all age groups (n=79, 69.9% and n=34, 30.1%, respectively), irrespective of the type of job (table 2).

Table 2 also describes the economic activity, ethnicity, mechanism of injury and activity taking place when the injury occurred for the injured women and men. For those women who had home injuries (n=86), 35 (40.7%) were home-makers and their most common home injuries were falls (n=40,460.5%), burns or scalds (n=15, 17.4%), cuts/piercings (n=11, 12.8%) and animal related (n=9, 10.5%). For men who had a home injury (n=55), their most common injuries were falls (n=28, 50.9%) and cuts/piercings (n=12, 21.8%).

### Costs of injury

The costs of injury and OOP expenditure incurred by the 139 injured persons are presented in table 3. The total mean cost (transport and treatment) of work-related injury was US$143.3 (SD 276.7) higher than for home injuries (US$130.4, SD 347.6). The mean OOP expenditure was higher for home injuries than work-related injuries (US$83.1, SD 199.6 and US$75.6, SD 127.7, respectively) and accounted for 57.9% of the total costs of injury.

**Table 3:** Total costs and out-of-pocket (OOP) payment (in US$)

	Home related (n=86) Mean (SD)	Work related (n=53) Mean (SD)
Treatment costs	122.7 (342.8)	128.4 (260.4)
Transport costs	7.7 (21.6)	14.9 (42.6)
Total costs	130.4 (347.7)	143.3 (276.8)
OOP expenditure	83.2 (199.8)	75.6 (127.8)

US$1=110 Nepalese rupees (June 2019).

Thirty-seven (26.6%) participants (22 home injuries and 15 work-related injuries) reported that these costs prohibited other HH expenses. Among those who reported such financial constraints 25/37 (67.6%) borrowed money for treatments. The mean amount of borrowed money for home-related and work-related injuries were US$294.2 (SD 371.6) and US$326.4 (SD 441.1), respectively. The mean treatment costs for these two groups were US$471.8 (SD 729.0) and US$398.7 (SD 483.4), respectively.

**Figure 1:**
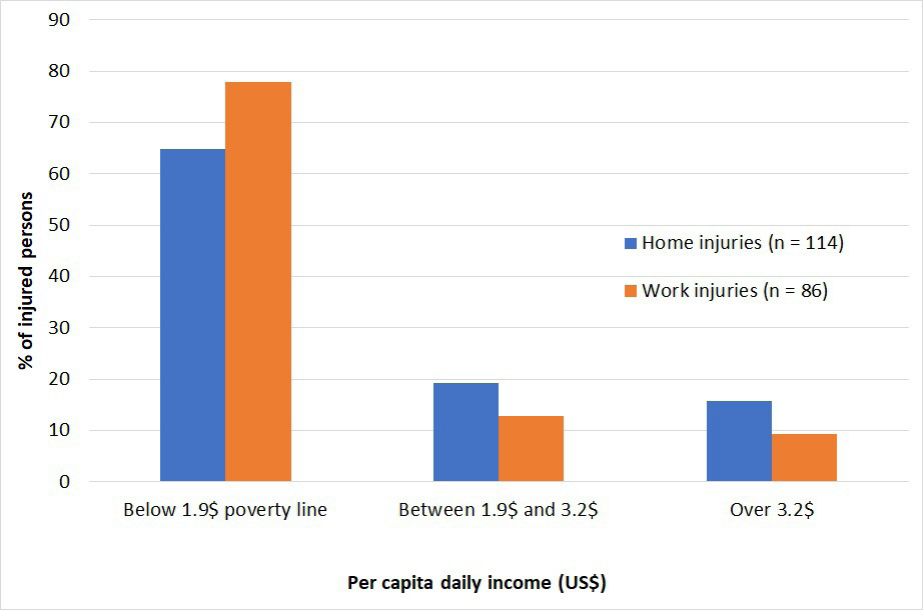
Distribution of injured persons by income group.

In our study, monthly income was reported by 200 (out of those 254 with home or work-related injuries) and 141 (70.5%) had an income of below US$1.9 per day (living below the World Bank poverty line).^13^ In Thaha, all of the injured persons (n=27) were in this category while 78.0% (n=50) in Bakaiya and 61.0% (n=123) in Hetauda were living below the poverty line. Among those with work-related injuries, 77.9% (n=67) were earning less than US$1.9 daily while this proportion was lower (64.9%, n=74) for those with home injuries. The results also suggest that both home and work-related injuries were higher among the low-income earners compared with those participants in the other two income brackets (p=3.98; df 1; Χ^2^=0.046) (figure 1).

## Discussion

To our knowledge, this is the first comprehensive study to identify, describe, and study distribution and inequalities of injuries related to home and work in Nepal. The survey data were collected from three different palikas that were typical of the Makwanpur district of Nepal. The findings suggest that there are particular at-risk groups for both home and work-related injuries. Out of all the palikas, the rural area (Bakaiya) had the highest rates of injuries, which confirms findings from other South Asian countries where rural areas also had high rates of injury.^14 15^ HH surveys previously conducted in different parts of Nepal recorded injury rates ranging between 7 per 1000 and 31 per 1000 per year.^4–7^ While these studies recorded minor and major injuries, none of them differentiated between home and work-related injuries.

High rates of home injuries in children in Nepal have previously been reported^9^ and have also been found in Bangladesh.^15^ Our study found that falls and cuts/piercings were the main mechanism for both home-related and work-related injuries. Fall injuries have also been the most commonly reported injuries in two studies in India^16 17^ and Pakistan^18^ where the children under the age of 15 years were at high risk of fall injuries. However, falls were found to be an equally serious problem for the elderly population in Bangladesh.^19^ Unlike Bangladesh, in our study we found falls were common among children aged 1–14 years (33.8%) and just 14.7% were among older people aged over 65 years. In our study, non-fatal burns and scalds were more common home injuries (14.2%) than in Bangladesh (<4%)^20^ but more similar to India (17%)^17^; they are a major cause of morbidity and disability.

In relation to home injuries, the percentage of all injuries that were home related in our study are similar to those reported in a recent systematic review conducted in Nepal.^10^ Work-related injuries were much higher in our study (28.7%) than in those studies included in the review (11%–19%).^10^ Earlier studies considered the place of injury occurrence but our study added context and applied operational definitions to the place of injury occurrence, for example, home or work related. The high number of work-related injuries could be explained by two reasons. First, the definition we used included unpaid work because it contributes to a family’s subsistence by providing an alternative to paid work for that family.^20^ For example, many families living in rural Nepal keep their own livestock and grow their own food. Second, as per the topographical and sociodemographic situation of the survey district, many people were engaged in manual and informal/unpaid work; the latter is common in rural areas. We also explored injuries in relation to economic activity, ethnicity and costs. In relation to agricultural injuries, our findings support those from an earlier Nepalese study where 69% of farmers reported an injury in the past 12 months.^21^

Our study found 6.2% of all work-related injuries were among children below 15 years. Although we categorised children sustained work-related injuries, as per our working definition, most of these injuries were associated with unpaid work while they tended the family livestock and undertook animal husbandry.

Nepal is a diverse country in respect of ethnicities with 125 ethnicities recorded in the last census.^22^ A previous study undertaken in the same district as our survey reported frequencies for emergency department (ED) attendance. They also reported that there was a higher proportion of Tamang and Brahmin ethnic groups who attended EDs with an injury.^23^ However, when rates of injury were calculated, we found that the Brahmin and Rai ethnicities had the highest rates of home and work-related injury, respectively. The previous study looked at ED attendance and reported frequencies whereas our study was a population survey and we report rates of injury. Tamang were the majority ethnic group in Makwanpur district and they had the highest absolute number of injuries^22^ but their rates of injury were lower than Rai and Brahmin groups. Most of the injured Rai ethnic group in our study live in the Bakaiya (rural) palika but this Rai community (Danuwar or Dewas Rai) is different to those who live in Eastern Nepal (Kirant Rai).^24^ In Nepal Tamang and Rai ethnic groups (Janjati) have been historically disadvantaged and the poverty is very high among them. So far, such groups are lagging behind in terms of income/assets, access to services and human development indicators.^25^ Rai (Dewas Rai) people traditionally live on fishing but have shifted their livelihoods to agriculture.^24^ In addition to agriculture and animal husbandry, daily wage labour is a livelihood for Tamangs of Makwanpur.^26^ The inequality in injury incidence by ethnic group suggests that this should be investigated further.

The costs of injuries in our sample were high in relation to income and were higher for work-related injuries than home injuries. To the best of our knowledge, there have been no previous studies in South Asia that have explored costs of home and work-related injuries.^27^ The World Bank estimated the rate of poverty (defined as an income of $1.90 per person per day) to be 8% in Nepal in 2019.^13^ In our study we asked the respondents about the family monthly income, this was then calculated per capita (US$). We found that the majority of home and work-related injuries happened to those who were living below US$1.9 per day. Taking loan (ie, from bank or cooperatives) or borrowing money (ie, from friends, neighbours and relatives) are the most common strategies for managing acute and catastrophic expenses. These strategies are common also because the uptake of health insurance scheme is quite low (around 5%) in Nepal.^28^

### Strengths and limitations

There are three principal strengths of this study, the first being the representative nature of the HHs that were recruited due to the sampling method used. Second, trained data collectors were committed and well supervised which led to complete data being collected with very few missing data. Third, HHs were offered over 100 different ethnic groups to choose from, thus making this the first comprehensive HH survey to explore injury by ethnic group in Nepal. Participants were willing to report their ethnic group and our findings indicate that there are inequalities by ethnic group that warrant further investigation. Although previous injury HH surveys provided information on the location of injury, there are no studies that reported home-related and work-related injuries. One limitation of our study was that the participants were asked to report non-fatal injuries that happened in the previous 3 months and 5 years for fatal injuries. While this was thought to be feasible for participants, there is a chance of recall bias as people tend to forget more minor injuries.^29^ A second limitation was the cross-sectional nature of this study so we cannot comment on causality. As in any observational study, the potential for unmeasured confounding exists and the nature of non-fatal injuries may have differed if it had been conducted at a different time of year. It is likely that there will be seasonal variation, for example, during the monsoon when travel is reduced and potentially more dangerous, there are monsoon-related injuries, such as drowning. A third limitation was that we had sociodemographic details just for those participants with an injury but not for those un-injured participants, therefore regression models to assess the association between the risk factors and injury could not be performed.

## Conclusion

Inequalities in injury were found in relation to rural living, age, sex, low income and specific ethnic groups. Falls and cuts/piercings were the main mechanisms of injury for both home-related and work-related injuries; non-fatal burns and scalds were common home injuries. This study highlights the need for the development and implementation of injury prevention programmes and policies that target geographical and sociodemographic differences in injury incidence and particular home-related and work-related injuries.

**What is already known on the subject**

- There is limited information about the distribution of injuries by ethnicity and income groups.
- Limited epidemiological data are available in Nepal about the location of injury, activity taking place when injury occurred and the costs of injury.

**What this study adds**

- People from rural areas experience more injuries than people living in urban or city areas.
- Manual workers sustain more work-related injuries than those in other occupations.
- Certain ethnic groups have higher rates of injuries than other ethnic groups.
- People with lower income experience more injuries than people with higher incomes.
- Injury costs are high in relation to average per capita income.

## Data Availability

Data are available upon reasonable request.
